# Bilateral Carotid-Cavernous Fistula: A Diagnostic and Therapeutic Challenge

**DOI:** 10.1177/23247096221094181

**Published:** 2022-06-24

**Authors:** Rohan Sharma, Christian Ponder, Mudassar Kamran, Joseph Chacko, Nidhi Kapoor, Krishna Mylavarapu, Sanjeeva Onteddu, Krishna Nalleballe

**Affiliations:** 1 University of Arkansas for Medical Sciences, Little Rock, USA; 2 Baptist Health Medical Center, Little Rock, AR, USA

**Keywords:** arteriovenous malformation, secondary headache disorders, ocular motility, carotid-cavernous fistula, endovascular transvenous coil embolization

## Abstract

Carotid-cavernous fistula (CCF) is an aberrant communication between the main trunk or branches of carotid artery and the cavernous sinus. Most of the cases of CCF occur following head trauma, but congenital and spontaneous cases have been reported. We report an interesting case of bilateral CCF with no history of trauma, thus most likely spontaneous form. Since it is rare, it was a diagnostic challenge. The suspicion of this diagnosis was made due to clinical features of headache, signs of increased Intracranial Pressure (ICP) (nausea, vomiting, and worsening headaches during Valsalva), exophthalmos, periorbital edema, periorbital erythema, chemosis, and conjunctival injection in both eyes. It was diagnosed with a 4-vessel angiography (digital subtraction angiography) which is the gold standard and was managed successfully with endovascular coil embolization.

## Case Presentation

A 74-year-old woman with history of chronic kidney disease and systemic hypertension presented with diplopia and headache for 3 weeks. She had developed blurred vision and intermittent diplopia, followed by severe bifrontal throbbing headache associated with nausea, vomiting, photophobia, that worsened with cough and Valsalva maneuver and improved briefly with over the counter pain medications. After a few days, she was seen by her primary care physician who treated her for suspected sinusitis with antibiotics; however, she had no significant improvement in her symptoms. She then presented to the emergency room (ER) for further evaluation. On presentation, she had blurred vision and binocular diplopia. She also had severe headache which did not abate with treatment given in the ER. She denied any recent illnesses, including cough, fever, diarrhea, or weight loss.

On exam, she was hypertensive to 182/73 mmHg; other vitals were within normal limits. She appeared anxious due to headache. Her neck was supple without any rigidity; heart rate was regular. Lungs were clear bilaterally, and abdomen was soft and nontender, without organomegaly. On neurological examination, she was awake, alert, and oriented to person, place, and time with intact language and speech. Strength and sensations were normal bilaterally. Finger-to-nose test was impaired due to visual impairment but heel-to-shin test was intact.

Uncorrected visual acuity was 20/70 in left eye and 20/100 in right eye. Visual field testing showed generalized constriction. The patient had a 5-mm pupil that constricted to 3 mm in the right eye and a 6-mm pupil that constricted to 4 mm in the left eye. Bilateral pupils were briskly reactive, without any relative afferent pupillary defect. The extraocular movements were restricted in all directions of gaze in both eyes. The patient had bilateral exophthalmos, periorbital edema, and periorbital erythema. The conjunctiva showed chemosis and conjunctival injection in both eyes (Figure 5A). The portable slit lamp examination was remarkable for dilated “corkscrew” episcleral vessels that extended to the limbus bilaterally and nuclear sclerotic cataracts bilaterally. Both the anterior chambers were deep and quiet and the iris was round and flat. Intraocular pressure (IOP) measured 27 mmHg in right eye and 24 mmHg in left eye. Dilated fundus examination revealed dilated retinal veinules bilaterally. The vitreous was clear, the optic nerves were pink and sharp, maculae were flat, and peripheral retinae were flat with no holes or tears noted bilaterally.

**Figure 1.**
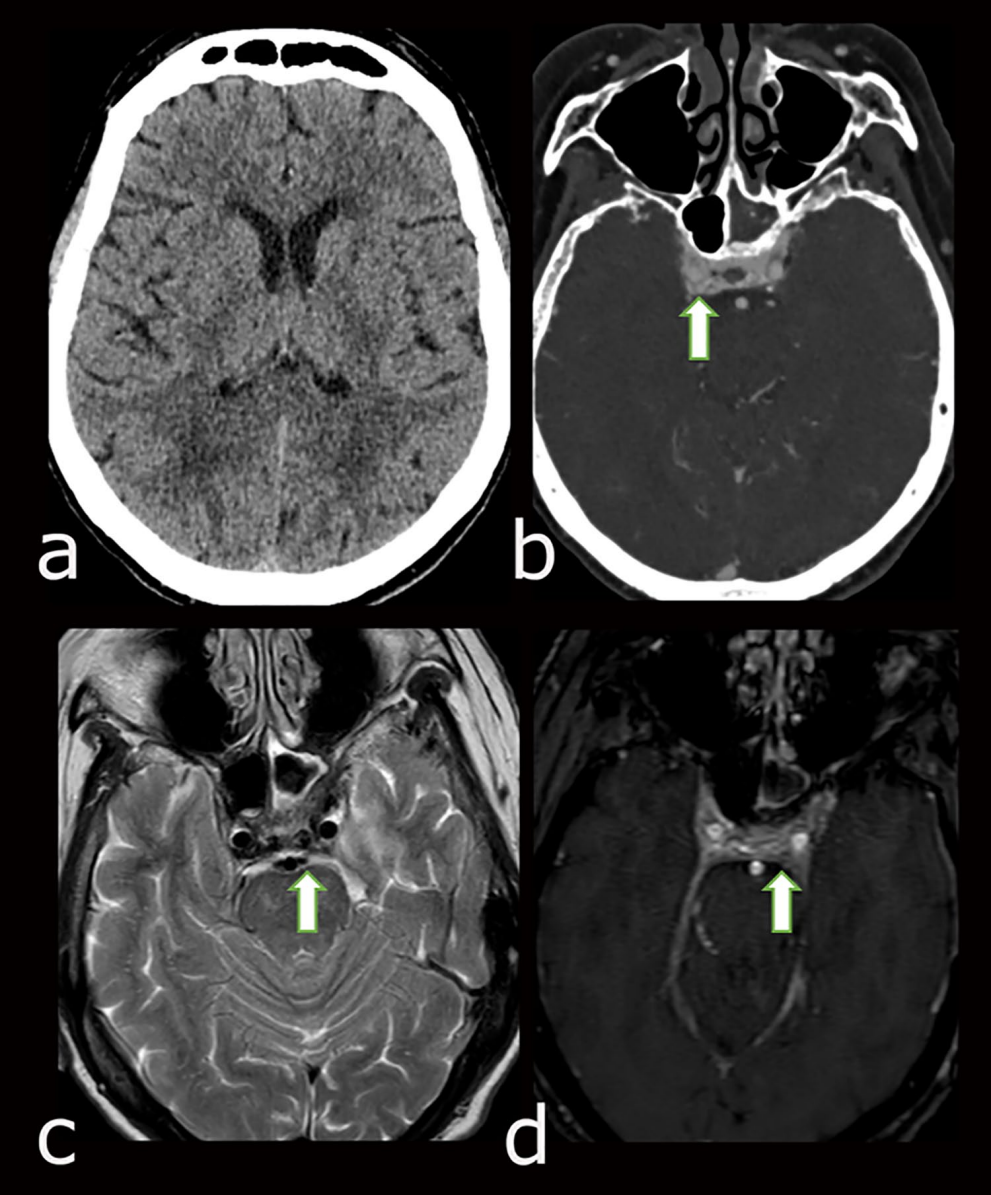
Cross-sectional imaging workup. (a) Initial non-contrast head CT excluded intracranial hemorrhage, enlarged ventricles, and intracranial mass; (b) CT angiogram demonstrates early enhancement of prominent cavernous sinuses and enlarged facial veins; (c) T2-weighted Magnetic Resonance (MR) image shows prominent flow voids in both cavernous sinuses suggestive of high blood flow; and (d) time-of-flight MR angiogram demonstrates flow signal in both cavernous sinuses.

Computreized Tomography (CT) head without contrast showed no intracranial hemorrhage, enlarged ventricles, and no intracranial mass (Figure 1A). CT angiogram of head demonstrated findings typical for carotid-cavernous fistula (CCF) that included prominent cavernous sinuses with convex margins, early enhancement of both cavernous sinuses, enlargement of both superior ophthalmic veins (SOVs), prominent angular and facial veins, proptosis, and mild retro bulbar fat stranding in both orbits (Figure 1B).

**Figure 2.**
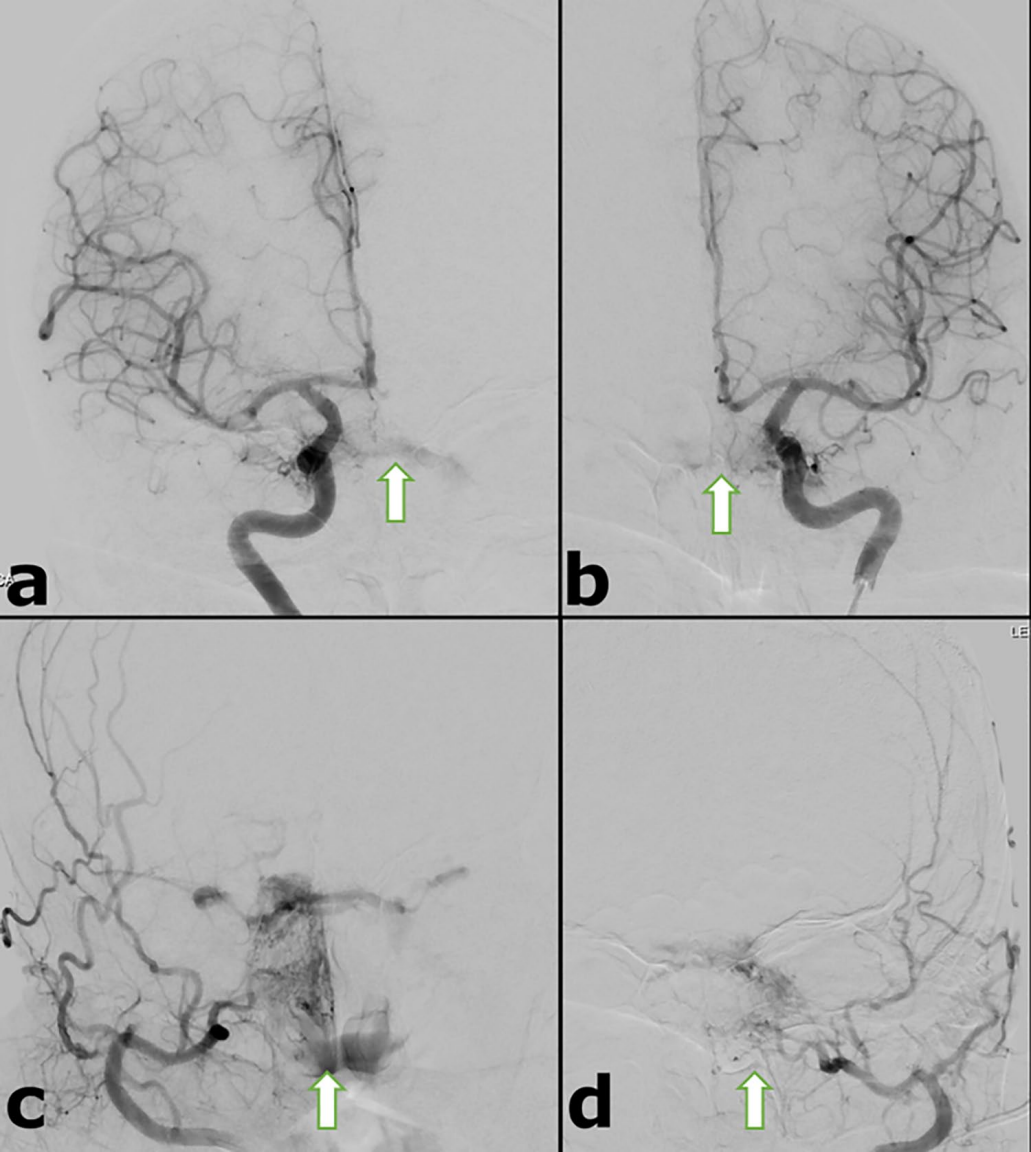
Pre-embolization selective catheter angiogram. (a and b) Right and left ICA injections demonstrate early filling of cavernous sinuses fed by the cavernous branches of both ICAs. (c and d) Right and left ECA injections demonstrate early filling of cavernous sinuses fed by the meningeal branches of both ECAs. Abbreviations: ICA, internal carotid artery; ECA, external carotid artery.

**Figure 3.**
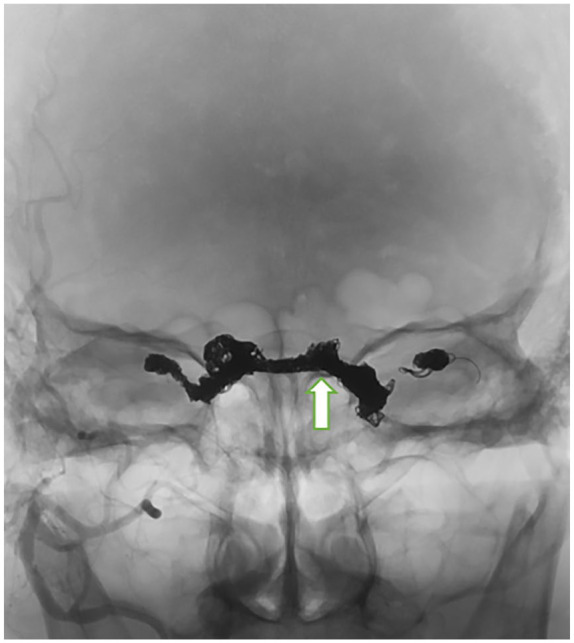
Frontal fluoroscopic images. Radiodense coils in both cavernous sinuses, both superior ophthalmic veins, and inter-cavernous sinus.

**Figure 4.**
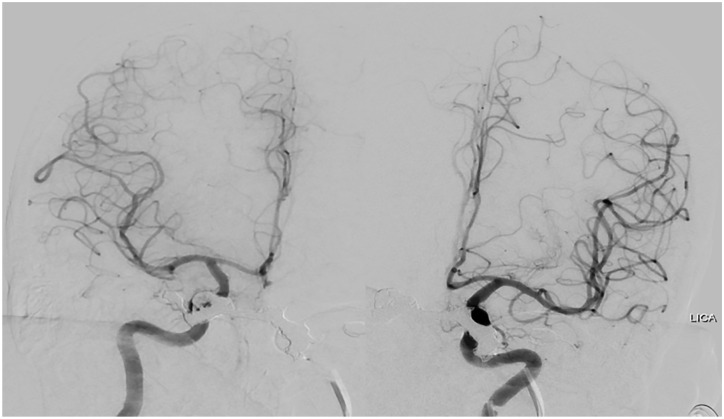
One-month post-endovascular coil embolization follow-up catheter angiogram. Right and left ICA injections demonstrate no residual early filling of the cavernous sinuses.Abbreviation: ICA, internal carotid artery.

T2-weighted MR image showed prominent flow voids in both cavernous sinuses suggestive of high blood flow with additional note made of focal tenting of the left posterior globe secondary to stretching of the left optic nerve (Figure 1C). Time-of-flight Magnetic Resonance (MR) angiogram demonstrated flow signal in both cavernous sinuses (Figure 1D). Catheter angiogram showed indirect (Barrow type D) bilateral CCFs fed by meningeal branches of both external carotid arteries and cavernous branches of both internal carotid arteries (Figure 2). Subsequent to multidisciplinary team discussion, the patient underwent endovascular treatment that comprised trans venous coil embolization of both cavernous sinuses and both SOVs using detachable coils (87 detachable coils, Figure 3). Postprocedural recovery was uncomplicated, and she was discharged home on postprocedure day 1. She had remarkable improvement of the exophthalmos, conjunctival chemosis and injection in both eyes (Figure 4B).

**Figure 5.**
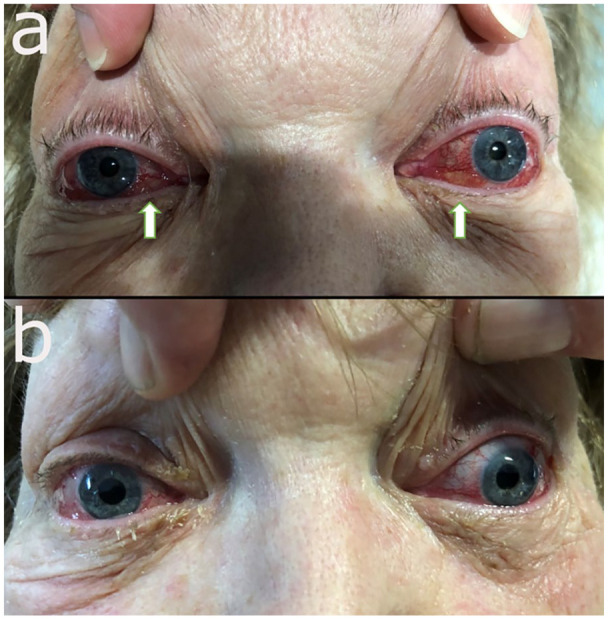
(a) Pre-embolization image demonstrates exophthalmos, periorbital edema and erythema, and conjunctival chemosis and injection in both eyes; (b) post-embolization day 1 images show remarkable improvement with resolution of the prior findings.

At 2-week follow-up in ophthalmology clinic, patient’s headache had improved and her conjunctival chemosis had completely resolved. Uncorrected visual acuity improved to 20/50 in the right eye and 20/40 in the left eye. Intraocular pressure improved to 14 mmHg in the right eye and 12 mmHg in the left eye. There was resolution of her dilated corkscrew episcleral vessels and her conjunctivae were not injected. She still had ophthalmoplegia and diplopia; however; there was subjective improvement when compared with the time of initial presentation. One-month follow-up catheter angiogram demonstrated complete resolution of CCFs, with no residual arteriovenous shunting (Figure 5).

## Discussion

Carotid-cavernous fistula is an aberrant communication between the main trunk or branches of carotid artery and the cavernous sinus. Majority of the cases of CCF occur following head trauma,^1^ but congenital^2^ and spontaneous^3^ cases have been reported. However, aneurysm subarachnoid hemorrhage, pregnancy, hypertension, atherosclerotic vascular disease, and connective tissue disorders have been associated with CCF.^4^ These shunts are anatomically divided into 4 categories. Type A are direct shunts between the internal carotid artery and the cavernous sinus and are high flow; type B are dural shunts that connect meningeal branches of the internal carotid artery to the cavernous sinus; type C are dural shunts connecting meningeal branches of the external carotid artery to the cavernous sinus; and type D are dural shunts between the meningeal branches of both internal and external carotid arteries and the cavernous sinus.^5^ Preechawat et al^6^ reported the incidence of type B, type C, and type D with 14%, 15%, and 71% shunts in 80 CCF patients, respectively.

Alexander et al^7^ found that the majority of patients with indirect CCF were females with mean age of 61 years, with mean duration from symptom onset to diagnosis of 234 days. Clinical signs and symptoms of CCF are conjunctival chemosis and injection, proptosis, ophthalmoplegia, pulsating exophthalmos, diplopia, orbital pain, bruits, increased IOP, and decreased vision.^1,6,8^ Audible bruit is appreciated on examination by auscultation over the closed eyelid. Our patient presented with all of the classic findings except pulsating exophthalmos and an audible bruit, likely because she had indirect low-flow fistulae. Since the clinical symptoms of CCF are often nonspecific and indolent, the diagnosis and treatment are often delayed as evident by the lengthy time from symptom onset to diagnosis mentioned above.

Intracranial hemorrhage or hemorrhagic infarcts are rare but dangerous complications of CCF and are associated with cortical venous reflux (CVR).^7,9,10^ Cortical venous reflux has been found to be more common in patients with diplopia, cranial nerve palsy, elevated IOP, chemosis, and bruit.^7^ Traditional 4-vessel angiography (digital subtraction angiography) is the gold standard to diagnose CCF and CVR, which may show tortuous and engorged veins, delayed appearance of the veins and focal staining, along the region of CVR in the venous phase of vertebral or internal carotid arteriography.^9^ Additional imaging clues to aid diagnosis include enlarged SOVs, thick extraocular muscles, enlarged cavernous sinus showing convexity of the lateral wall on brain CT and Magnetic Resonance Imaging (MRI), increased signal intensity of the brain parenchyma in the region and CVR on brain MRI, and decreased cerebral blood flow on the region of CVR on single-photon emission computerized tomography (SPECT).^9^

Exophthalmos with conjunctival chemosis secondary to CCF can be confused for Graves’ exophthalmos, especially in bilateral disease such as our patient.^11^ Other diseases such as orbital pseudotumor, orbital vasculitis secondary to granulomatosis with polyangitis, polyarteritis nodosa, Tolosa-Hunt syndrome, and intracranial sarcoidosis may mimic CCF symptoms.^1^ The majority of CCFs are usually not life-threatening. However, they can cause serious debility if they become symptomatic. Spontaneous resolution of CCF is rare, and surgical intervention should be considered if patients develop signs of elevated IOP, exophthalmos or proptosis, diminution of vision, ophthalmoplegia, or unrelenting headache.^1,4,6,12^ Another important guide for intervention is presence of CVR. Bulters et al^13^ found that the untreated dural arteriovenous fistulae which had CVR showed a 13% annual incidence of hemorrhage after diagnosis was made. Several different treatment modalities exist for the treatment of CCF including direct surgery, stereotactic radiosurgery, intermittent manual self-compression of the affected internal carotid artery with the contralateral hand, conventional radiation therapy, and endovascular intervention through trans-arterial or trans venous routes.^4,6,10,12-14^ Endovascular interventions are safe and effective in treatment.^4,6,8,14,15^ There remains, however, a risk of complications with any intervention including arterial dissection, bleeding and hematoma formation, complete ophthalmoplegia, visual acuity loss due to a central retinal artery or vein obstruction, intracranial hemorrhage, and cerebral infarction.^1,6,14^

## Conclusion

Our patient had bilateral CCF. She had no history of recent or prior trauma and we believe she may have had a spontaneous form. For patients presenting with diplopia, exophthalmos and chemosis CCF must be considered in the differential.
